# EGCG Prevents the Transcriptional Reprogramming of an Inflammatory and Immune-Suppressive Molecular Signature in Macrophage-like Differentiated Human HL60 Promyelocytic Leukemia Cells

**DOI:** 10.3390/cancers14205065

**Published:** 2022-10-16

**Authors:** Celia Kassouri, Sahily Rodriguez Torres, Narjara Gonzalez Suarez, Stéphanie Duhamel, Borhane Annabi

**Affiliations:** 1 Laboratoire d’Oncologie Moléculaire, Département de Chimie, and CERMO-FC, Université du Québec à Montréal, Montreal, QC H2X 2J6, Canada; 2 Goodman Cancer Institute, McGill University, Montreal, QC H3A 1A3, Canada

**Keywords:** EGCG, inflammation, immunity, leukemia, macrophage

## Abstract

**Simple Summary:**

Scientists are increasingly harnessing the power of the immune system to prevent cancer. While macrophages are a major component of the tumor microenvironment where they orchestrate various aspects of immunity, dysregulated immune and inflammatory responses will play a key role in cancer initiation and progression. Depending on their activation status, macrophages can have a dual impact on tumorigenesis by either antagonising cytotoxic immune cells or enhancing antitumor responses. Increased infiltration of tumor-associated macrophages has long been associated with poor patient prognosis in most solid cancers, highlighting their value as potential diagnostic and prognostic biomarkers in cancer. A variety of macrophage-centered approaches to cancer therapy have been investigated, including strategies to prevent tumor-promoting activities. In this study, we highlight the chemopreventive properties of EGCG derived from green tea that underpin its ability to re-program macrophage molecular signature and function.

**Abstract:**

Background: The promyelocytic leukemia cell differentiation process enables recapitulation of the polarized M1 or M2 macrophage-like phenotype with inflammatory and immune-suppressive properties. While evidence supports the anti-inflammatory effect of dietary-derived epigallocatechin-3-gallate (EGCG), its impact on the onset of immune phenotype molecular signature remains unclear. Methods: Human HL60 promyelocytic cells grown in suspension were differentiated into CD11b^High^/CD14^Low^ adherent macrophages with phorbol 12-myristate 13-acetate (PMA). Gelatin zymography was used to assess the levels of matrix metalloproteinase (MMP)-9, and total RNA was isolated for RNAseq and RT-qPCR assessment of differentially expressed gene levels involved in inflammation and immunity. Protein lysates were used to assess the phosphorylation status of signaling intermediates involved in macrophage-like cell differentiation. Results: Cell adhesion and induction of MMP-9 were indicative of HL60 cell differentiation into a macrophage-like phenotype. The extracellular signal-regulated kinase (ERK), glycogen synthase kinase (GSK)-3, p90 ribosomal S6 kinases (RSK), and cAMP-response-element-binding protein (CREB) were all phosphorylated, and EGCG reduced such phosphorylation status. Increases in inflammation and immunity genes included, among others, *CCL22*, *CSF1*, *CSF2*, *IL1B*, and *TNF*, which inductions were prevented by EGCG. This was corroborated by unbiased transcriptomic analysis which further highlighted the capacity of EGCG to downregulate the hematopoietic stem cell regulator *CBFA2T3*. Conclusion: EGCG inhibits inflammatory signaling crosstalk and prevents the onset of an immune phenotype in macrophage-like differentiated cells.

## 1. Introduction

Chronic inflammatory processes affect all stages of tumor development and therapy efficacy [1,2]. The signaling crosstalk that coordinates the tumor-promoting and tumor-antagonizing effects of inflammation, and the molecular signature linking immune and inflammatory processes unfortunately, still require a better understanding [3]. In addition, the interaction between immunological and cancer cells as well as the function of tumor-associated macrophages (TAMs) in the tumour microenvironment is complicated [4]. TAMs, among the different immune cells, are highly prevalent within the tumour’s microenvironment and play a significant role in promoting tumour growth [5,6]. TAM-induced inflammatory responses are important for tumour growth at various stages. The relationship between inflammation, the immune system, and carcinogenesis is mediated by a variety of cytokines produced by TAMs and other immune cells in the tumour microenvironment [7,8].

Leukemia is a cancer of the blood-forming tissues that affects the blood and bone marrow, where acute myeloid leukemia (AML) comprises nearly 80 percent of all adult acute leukemias [9]. Despite significant improvements in leukemia management and diagnosis, inflammatory response mediating its crosstalk with immune function reveals clinical features in AML, which still require new therapeutic options [10]. Among those, monoclonal antibodies-based immunotherapies have recently emerged and appear to represent the future of AML treatment [11]. Here, the use of a tumorigenic HL60 cell line, isolated from the peripheral blood tissue of a 36-year-old female patient [12], has proven to be a potent model for studies of human myeloid cell differentiation and differentiation in general [13]. In addition, the HL60 xenograft model is also a relevant preclinical model enabling screening studies of AML chemotherapeutics and anticancer prodrugs [14].

It is possible to trigger the differentiation of HL60 promyelocytic cells into neutrophils. Studies have demonstrated that estrogens can regulate the expression of genes related to the immunological and inflammatory response, such as chemokine and cytokine genes involved in neutrophil recruitment and activation in normal as well as pathological settings [15,16]. When HL60 cells are treated with the protein kinase C (PKC) activator phorbol 12-myristate 13-acetate (PMA), a macrophage-like phenotype can also be attained [17,18]. It is interesting to note that MMP-9 expression, which is connected to myeloid cell differentiation, inflammation, and angiogenesis processes, increases in response to PMA-mediated HL60 differentiation [19]. The expression of MMP-9 gene and protein and of the mRNA stabilizing factor Human antigen R (HuR) were inhibited upon epigallocatechin-3-gallate (EGCG) treatment and such transcriptional regulation is also observed in the inhibition of human brain microvascular endothelial cells’ three-dimensional in vitro tubulogenesis [20].

Recent studies have shown that diet-derived plant constituents, the majority of which fall under the chemical categories of alkaloids, coumarins, flavonoids, polyphenols, and terpenoids, have anti-inflammatory properties [21]. The recruitment and infiltration of macrophages into the tumour, where the microenvironment subsequently stimulates them to support the malignant growth of cancer cells, are unknown effects of flavonoids, despite their usage in targeting inflammatory pathways [22]. EGCG was reported to indirectly inhibit infection by regulating immune inflammation and anti-oxidation [3]. Given the selective biomarker targeting of EGCG, we first questioned at the transcriptomic level how oncogenic-mediated differentiation of HL60 promyelocytic leukemia cells into a macrophage-like phenotype could impact their immune and inflammatory phenotype. Then, we explored the capacity of diet-derived EGCG to prevent protein phosphorylation intermediate status in adherent HL60 cells upon differentiation by PMA and how this subsequently altered the immune and inflammatory phenotype of macrophage-like cells.

In the present study, we characterized the transcriptomic reprogramming of the responsive genes involved in the crosstalk between immune and inflammation processes in macrophage-like terminally differentiated human HL60 promyelocytic leukemia cells and evidenced how EGCG alters such molecular signature. The phosphorylation status of signal transducing intermediates was also analyzed in adherent macrophage-like cells and supports the molecular rationale for pleiotropic diet-derived chemoprevention strategies.

## 2. Materials and Methods

### 2.1. Materials

Sodium dodecyl sulfate (SDS) and bovine serum albumin (BSA) were purchased from Sigma-Aldrich Corp (St Louis, MO, USA). Cell culture media was from Life Technologies Corp (Carlsbad, CA, USA). Electrophoresis reagents were purchased from Bio-Rad Laboratories (Hercules, CA, USA). The HyGLO™ Chemiluminescent HRP (horseradish peroxidase) Antibody Detection Reagents were from Denville Scientific Inc. (Metuchen, NJ, USA). Micro bicinchoninic acid (BCA) protein assay reagents were from Pierce (Micro BCA™ Protein Assay Kit; Thermo Fisher Scientific, Waltham, MA, USA). The polyclonal antibodies against extracellular signal-regulated kinase (ERK), phosphorylated (P)-ERK, Glycogen synthase kinase-3 alpha and beta (GSK3-α/β), phosphorylated (P)-GSK3-α/β (Ser21/9), C-AMP Response Element-binding protein (CREB), phosphorylated (P)-CREB (Ser133), and the monoclonal antibody against the 90 kDa ribosomal S6 kinases (RSK1/RSK2/RSK3), phosphorylated p90RSK (ser380) were all purchased from Cell Signaling Technology Inc (Danvers, MA, USA). Monoclonal Antibody against Human mitogen- and stress-activated protein kinase 1 (MSK 1), phosphorylated (P)-MSK1 and MSK2 (MSK1 S376, MSK2 S360) were purchased from R&D Systems Inc (Minneapolis, MN, USA). HRP-conjugated donkey anti-rabbit and anti-mouse immunoglobulin (Ig) G secondary antibodies were from Jackson ImmunoResearch Laboratories (West Grove, PA, USA). EGCG was from MP Biomedicals (Santa Ana, CA, USA). All other reagents were from Sigma-Aldrich Corp.

### 2.2. Cell Culture

The HL60 promyelocytic cell line was purchased from American Type Culture Collection (Manassas, VA, USA) and was kept in Iscove’s Modified Dulbecco’s Medium (Life Technologies Corp) containing 20% (*v*/*v*) fetal bovine serum (FBS) (HyClone^TM^; Thermo Fisher Scientific), 2 mM glutamine, 100 units/mL penicillin, and 100 μg/mL streptomycin (Wisent Inc., Quebec, QC, Canada). Upon thawing/resuscitating the HL60 cells from frozen vials, they were then passaged two times prior to treatments. The induction of macrophage differentiation was accomplished using the tumor-promoting phorbol-12-myristate-13-acetate (PMA) [23,24]. We wish to emphasize that, throughout the text, the “HL60 macrophage differentiation” condition represents the adherent subpopulation of HL60 cells that are immediately harvested after PMA treatment. This is important since numerous protocols can be found in the literature that use PMA to differentiate resting HL60 cells into “macrophage-like cells” (upon 2 and 8 days with various PMA concentrations, alone or in combination with other molecules). These adherent cells were then kept in culture for an additional 24–48 h and are referred to as “terminally differentiated macrophages” in this paper. Treatments of the cells with PMA or EGCG in a media from which FBS was withdrawn are referred to as cell starvation.

### 2.3. Total RNA Isolation, cDNA Synthesis, and Real-Time Quantitative PCR

Total RNA was extracted from cell monolayers using 1 mL of TriZol reagent for a maximum of 3 × 10^6^ cells as recommended by the manufacturer (Life Technologies, Gaithersburg, MD, USA). For cDNA synthesis, 1–2 µg of total RNA was reverse-transcribed using a high-capacity cDNA reverse transcription kit (Applied Biosystems, Foster City, CA, USA) or, in the case of the gene array: R2 First Strand kit (QIAGEN, Valencia, CA, USA). The cDNA was stored at −80 °C prior to PCR. Gene expression was quantified by real-time quantitative PCR using iQ SYBR Green Supermix (Bio-Rad, Hercules, CA, USA). DNA amplification was carried out using an Icycler iQ5 (Bio-Rad) and product detection was performed by measuring the binding of the fluorescent dye SYBR Green I to double-stranded DNA. The following primer sets were from QIAGEN: CSF-1 (Hs_CSF1_1_SG, QT00035224), CSF-2 (Hs_CSF2_1_SG, QT00000896), MMP-9 (Hs_MMP9_1_SG, QT00040040), GAPDH (Hs_GAPDH_1_SG, QT00079247) and Peptidylprolyl Isomerase A (PPIA) (Hs_PPIA_4_SG, QT01866137). The relative quantities of target gene mRNA were normalized against internal housekeeping genes PPIA and GAPDH. The RNA was measured by following a ∆C_T_ method employing an amplification plot (fluorescence signal vs. cycle number). The difference (∆C_T_) between the mean values in the triplicate samples of the target gene and the housekeeping genes was calculated with the CFX manager Software version 2.1 (Bio-Rad) and the relative quantified value (RQV) was expressed as 2^−∆CT^.

### 2.4. Total RNA Library Preparation

In order to prepare the libraries, total RNA (500 ng) was isolated from HL60 cell cultures. All samples had an RNA integrity number (RIN) above eight when RNA quality control was evaluated using the Bioanalyzer RNA 6000 Nano assay on the 2100 Bioanalyzer system (Agilent Technologies, Mississauga, ON, Canada). Using the KAPA mRNA-Seq HyperPrep kit (KAPA, Cat no. KK8581), the libraries were prepared. Illumina dual-index UMI was used for ligation, and cDNA libraries were required to be amplified using 10 PCR cycles. By using the QuBit and BioAnalyzer DNA1000, libraries were quantified. All libraries were diluted to 10 nM, and the KAPA library quantification kit was used for qPCR normalization (KAPA; Cat no. KK4973). Libraries were pooled to equimolar concentrations. There were three biological replicates.

### 2.5. RNA Sequencing

High RNA quality was confirmed as mentioned above, and samples were sequenced at the Genomics Core Facility of the Institute for Research in Immunology and Cancer (IRIC, Montreal, QC, Canada) using the Illumina NextSeq500 sequencer.

### 2.6. Reads Alignment and Differential Expression Analysis

Using the STAR aligner (STAR 2.7.1a), reads were aligned and sorted by coordinates to the human genome build 38 (GRCh38.p13) with version 37 of Gencode gene annotations [25,26]. STAR carried out gene quantification during alignment. The R packages DESeq2 (v 1.30.1) were used to find the genes that were differentially expressed between groups [27]. Only genes with adjusted *p*-values (adjp) < 0.05 and log2 fold changes > 1.0 were deemed significant after analysis. The significantly modulated gene expression values referred to as adjusted *p*-values (adjp) < 0.05 and log2 fold changes > 1.0 top were extracted from these studies, and the average expression values were used to draw the heatmap using the ggplot2 package in R version 3.35 [28]. Differences were deemed statistically significant for all statistical analyses if the adjp determined by the Student’s *t*-test with Bonferroni adjustment was <0.05.

### 2.7. Gene Set Enrichment Analysis

The gene set enrichment analysis (GSEA) was performed using the GSEA software version 4.2.3 [29] with the complete set of normalized input values, using the Hallmark, canonical pathway gene sets (chemical and genetic perturbations, BioCarta, Reactome, and Kegg), and Gene Ontology (GO) gene sets (Biological process). Molecular Signatures Database (MSig-DB), version 7.5.1 was applied to genes modulated in differentiated cells treated with EGCG (HL60 co-treated with PMA and EGCG) compared to undifferentiated cells (HL60 cultured in suspension) (values (adjp) < 0.05, and log2 fold change (FC) ≥ 1.0 were considered as significant) to generate a signature list of the top modulated gene signatures from the following Curated gene sets (C2CP and C2CGP) and Gene Ontology (C5). For all statistical analyses, differences were considered statistically significant if the adjp calculated by Student’s *t*-test with Bonferroni correction were <0.05. RNA-Sequencing of the HL60 cell lines.

### 2.8. Human Cancer Inflammation and Immunity Crosstalk PCR Array

The RT^2^ Profiler^TM^ PCR Array for Human Cancer Inflammation and Immunity Crosstalk (PAHS-181Z) was used according to the manufacturer’s protocol (QIAGEN). The detailed list of the key genes assessed can be found on the manufacturer’s website (https://geneglobe.qiagen.com/us/product-groups/rt2-profiler-pcr-arrays; accessed on 13 January 2022). Using real-time quantitative PCR, we reliably analyzed the expression of a focused panel of genes related to the inflammatory response, including some of the cancer-associated adipocyte markers already published. Relative gene expression was calculated using the 2^−∆∆CT^ method (“delta-delta” method), in which C_T_ indicates the fractional cycle number where the fluorescent signal crosses the background threshold. This method normalizes the ∆C_T_ value of each sample using five housekeeping genes (B2M, HPRT1, RPL13A, GAPDH, and ACTB). The normalized FC values are then presented as average FC = 2 (average ^∆∆C^_t_. The resulting raw data were then analyzed using the PCR Array Data Analysis Template (http://www.sabiosciences.com/pcrarraydataanalysis.php; accessed on 5 June 2022). This integrated web-based software package automatically performs ∆∆C_T_-based FC calculations from the uploaded raw thresholded cycle data.

### 2.9. Western Blot

Proteins (10–20 μg) were separated by SDS–polyacrylamide gel electrophoresis (PAGE) after cells were lysed in a solution containing 1 mM of each of NaF and Na_3_VO_4_. Proteins were then electro-transferred to polyvinylidene difluoride membranes and blocked with 5% nonfat dry milk in Tris-buffered saline (150 mM NaCl, 20 mM Tris-HCl, pH 7.5) containing 0.3% Tween-20 for an hour at room temperature (TBST; Bioshop, TWN510-500). TBST was used to wash the membranes, and they were then incubated with the appropriate primary antibodies (1/1000 dilution) in TBST that also contained 3% BSA and 0.1% sodium azide (Sigma-Aldrich) overnight at 4 °C. The membranes were incubated with horseradish peroxidase-conjugated anti-rabbit or anti-mouse IgG at 1/2500 dilutions for 1 h in TBST containing 5% nonfat dry milk after three TBST washes.

### 2.10. Gelatin Zymography

As previously mentioned, gelatin zymography was utilized to determine the level of pro-MMP-9 gelatinolytic activity [19]. In summary, a sample (20 μL) of the culture media was electrophoresed on a gel containing 0.1 mg/mL gelatin, a substrate that pro-MMP-9 is highly effective at hydrolyzing. The gels were then washed with nanopure distilled water after being incubated in 2.5% Triton^TM^ X-100. The gels were then stained with 0.1% Coomassie Brilliant Blue R-250 and destained in 10% acetic acid and 30% methanol in water. The gels were then further incubated at 37 °C for 20 h in a solution of 20 mM NaCl, 5 mM CaCl_2_, 0.02% Brij^®^-35, and 50 mM tris(hydroxymethyl)aminomethane (Tris)-HCl buffer, pH 7.6. Gelatinolytic activity was detected as unstained bands on a blue background.

### 2.11. Statistical Data Analysis

Data and error bars were expressed as mean ± standard error of the mean (SEM) of three or more independent experiments unless otherwise stated. Hypothesis testing was conducted using the Kruskal–Wallis test followed by a Dunn Tukey’s post-test (data with more than three groups) or a Mann–Whitney test (two-group comparisons). Probability values of less than 0.05 (*) or 0.01 (**) were considered significant and denoted in the figures. All statistical analyses were performed using the GraphPad Prism version 7 software (San Diego, CA, USA).

### 2.12. Code Availability

An R package ggplot2 wrapper for enhanced data visualization functionalities and code for dot plot visualization can be found at https://github.com/xmc811/ and https://ggplot2.tidyverse.org, respectively (accessed on 4 January 2022).

## 3. Results

**Figure 1:**
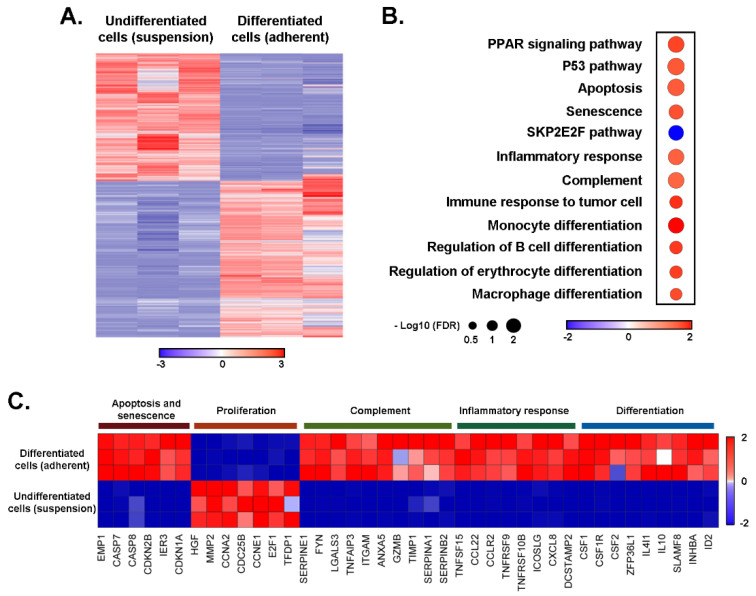
Transcriptomic analysis reveals the induction of differentiation and immune function genes upon macrophage-like differentiation of promyelocytic HL60 cells. Total RNA was extracted from HL60 cells cultured in suspension or PMA-treated adherent cells from three independent experiments and subjected to RNA-Sequencing. (**A**) Unsupervised hierarchical clustering of all the genes differentially expressed in pairwise comparison was tested with adjusted *p* value ≤ 0.05 and log2 fold change ≥ 1.0. (**B**) Dot plot showing changes in the normalized enrichment score for the most enriched pathways in PMA-treated adherent HL60 cells when compared to HL60 cultures in suspension. *p* value ≤ 0.05 and false discovery rate (FDR) *p* value ≤ 0.25. The top up- and down-regulated gene signatures of Hallmark, Gene Ontology, Genetic and chemical perturbations, and Canonical pathways. (**C**) Heatmap representation of the relative expression of known phenotypic markers for each of the indicated cellular processes.

Unbiased transcriptomic analysis reveals the induction of differentiation and immune activation programs upon macrophage-like differentiation of promyelocytic HL60 cells. In order to gain insight into the differential transcriptomic regulation, which occurs upon PMA-mediated HL60 differentiation into adherent macrophage-like cells, total RNA was isolated from suspension and adherent cell cultures and RNA-Sequencing was performed as described in the Methods section. Unsupervised hierarchical clustering revealed that PMA-differentiated adherent macrophage-like cells clearly displayed a gene expression signature distinct from that of undifferentiated HL60 cells cultured in suspension (Figure 1A). To gain further insight into these differences, we performed GSEA by comparing transcript expression levels from the differentiated macrophage-like cells to HL60 cell suspension cultures. Among those processes found to be significantly induced in terminally differentiated adherent cells, these included macrophage and monocyte differentiation, B cell regulation, inflammatory response, immune response to tumor cells, apoptosis, and senescence (Figure 1B, red dots). This was associated, among other genes, with a significant increase in the levels of differentiation genes CSF1, CSF2, and ID2 (Figure 1C).

PMA treatment of HL60 cells also induced an inflammatory transcriptional program associated with an enhanced complement activation signature (Figure 1B). This was corroborated by the increase in Granzyme B (*GZMB*) and the induction of the pro-inflammatory cytokines *TNFSF15*, *TNFRSF9*, and *CCL2*, and by the chemokine *CXCL8* transcripts (Figure 1C). In addition, GSEA showed significant enhancement for PPAR and p53 pathways, as well as apoptosis and senescence gene signatures. In agreement, increased levels of Caspases (*CASP7* and *CASP8*) and of the Cyclin-dependent kinase (CDK) inhibitors *C*DKN2B** and *CDKN1A* were observed in the PMA-differentiated HL60 cells (Figure 1C). Of particular interest, the *CDKN2B* gene encoding the tumor suppressor p15^INK4B^ is a critical regulator of senescence and apoptosis by acting as an inhibitor of the CDK that regulates progression through the G1 phase of the cell cycle [30,31]. *CDKN2B* is also essential for hematopoietic stem cell renewal [32].

Negative enrichment in SKP2E2F gene signature was observed upon cell differentiation (Figure 1B, blue dot). This was corroborated by the repression of the transcription factor E2F which is an essential regulator of cell cycle progression from G1 into the S phase, and which is regulated through phosphorylation by the CDK [33]. Accordingly, *E2F1* and CyclinA2 and E1 (*CCNA2* and *CCNE1*) transcripts were significantly reduced in the PMA-treated cells (Figure 1C). In conclusion, PMA-mediated differentiation into a macrophage-like phenotype appears to reduce the cell proliferation index and supports the induction of a differentiation program accompanied by increased immune functions.

**Figure 2:**
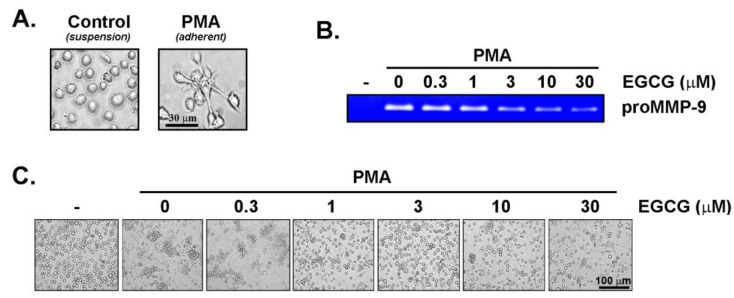
EGCG inhibits PMA-mediated human promyelocytic HL60 cell adhesion and MMP-9 secretion. Human HL60 cells were passaged in suspension as described in the Methods section and then treated with 0.6 μM PMA for 24 h in serum-free media. (**A**) Representative phase contrast pictures were taken of cells in suspension (**left**) and in adhesion (**right**). (**B**) Conditioned media was collected from serum starved HL60 cells in suspension and upon PMA treatment in the presence of increasing doses of EGCG. Gelatin zymography was performed to assess the levels of proMMP-9 secretion as described in the Methods section. (**C**) Representative phase contrast pictures were taken of cells treated or not with PMA, and in the presence of increasing concentrations of EGCG.

EGCG inhibits PMA-mediated human promyelocytic HL60 cell adhesion and MMP-9 secretion. The human HL60 promyelocytic differentiation process into an adherent macrophage-like phenotype was further validated. PMA treatment of serum-starved HL60 cells cultured in suspension efficiently led to an adherent cell phenotype (Figure 2A). This was associated with the induction of pro-MMP-9 secretion, in agreement with previous studies [17,18], where the acquisition of a CD11b^High^/CD14^Low^ macrophage phenotype was confirmed [34]. Interestingly, the co-treatment with increasing concentrations of EGCG was found to both inhibit PMA-induced MMP-9 secretion (Figure 2B) and to reverse the adhesive phenotype of the cells (Figure 2C). Altogether, this confirms that our cell differentiation protocol is functional and prompts for the subsequent investigation of the cell signaling intermediates involved in the acquisition of the macrophage/monocyte-like phenotype.

**Figure 3:**
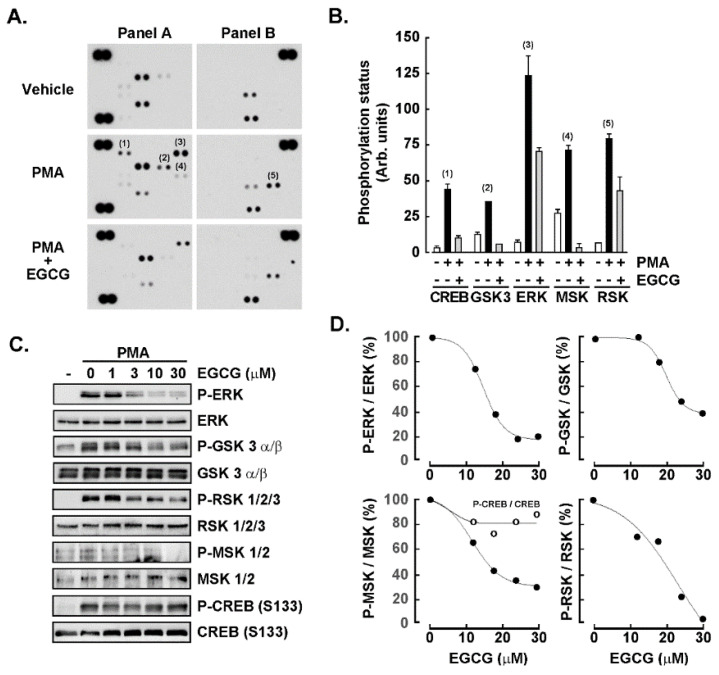
The induction of GSK3α/β, CREB, ERK1/2, and RSK1,2,3 phosphorylation status upon PMA-mediated human promyelocytic HL60 cell treatment is inhibited by EGCG. Protein lysates from human HL60 cells, either in suspension or treated with PMA were harvested and processed for (**A**) PhosphoScan pathway screening according to the manufacturer’s protocol (Representative autoradiograms are presented), and (**B**) quantified by scanning densitometry. (**C**) Protein lysates were harvested from untreated HL60 cells in suspension and adherent cells upon PMA treatment in the presence of increasing concentrations of EGCG. Representative autoradiograms are shown for the indicated total or phosphorylated signaling intermediates, whereas (**D**) scanning densitometry was performed to quantify each of the phosphorylated status levels. The uncropped blots are shown in File S1.

The induction of GSK3α/β, CREB, ERK1/2, and RSK1/2/3 phosphorylation status upon PMA-mediated human promyelocytic HL60 cell treatment is inhibited by EGCG. A screen of the potential phosphorylated signal transducing intermediate status was next performed using PathScan pathway as described in the Methods section. Protein lysates from human HL60 cells either in suspension, treated with PMA or with a combination of PMA and EGCG were harvested and processed. Autoradiograms from a representative experiment are shown (Figure 3A), and the intensity of significantly modulated phosphorylated intermediates was identified and quantified by scanning densitometry (Figure 3B). The signaling pathways which appeared to be both induced by PMA and which induction was prevented by EGCG in the screen were the cAMP-response-element-binding protein (CREB), the glycogen synthase kinase-3 (GSK3)α/β, the extracellular signal-regulated protein kinase (ERK)1/2, the mitogen- and stress-activated protein kinases (MSK) 1/2, and the p90 ribosomal S6 kinase (RSK) 1/2/3. Among those signaling intermediates whose phosphorylation status was not induced upon PMA treatment were FGR, mitogen-activated protein kinase 14 (P38α), c-Jun N-terminal kinase (JNK) 1/2/3, platelet-derived growth factor receptor β, Signal transducer and activator of transcription 5 α/β, P53, heat shock protein 60 (not shown). To further validate the respective inductions and EGCG inhibition observed, the HL60 suspension cells were either untreated, treated with PMA, or treated with PMA and increasing doses of EGCG. Protein lysates were harvested, and representative autoradiograms were shown for the indicated total or phosphorylated signaling intermediates (Figure 3C). Finally, the EGCG-mediated inhibition of the phosphorylation status of MSK, RSK, ERK, and GSK was confirmed, however, only very modest inhibition by EGCG was observed for CREB (Figure 3D).

**Figure 4:**
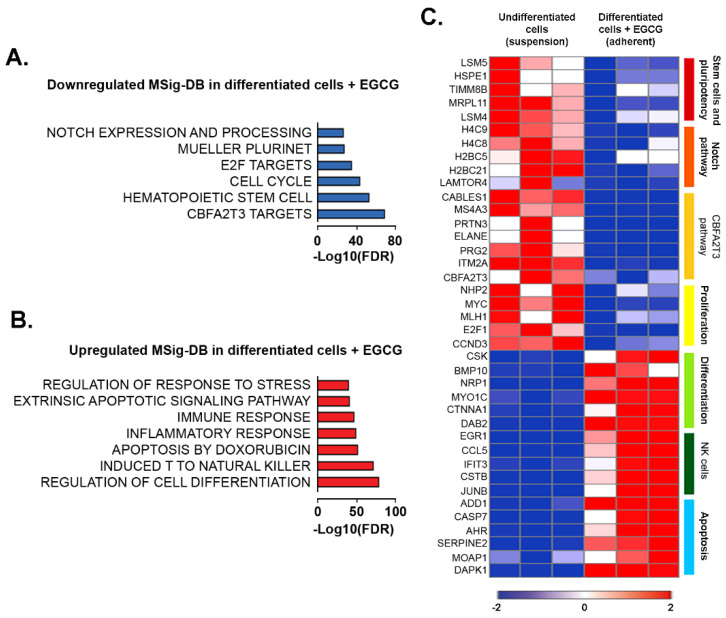
Transcriptomic analysis reveals the reduction in proliferation and stemness properties in macrophage-like promyelocytic HL60 cells in response to EGCG. (**A**,**B**) RNA from HL60 cells cultured in suspension or treated with PMA and EGCG for 18 h from three independent experiments was extracted and subjected to RNA-Sequencing. All differentially expressed genes in pairwise comparison, tested with adjusted *p* value ≤ 0.05 and log2 fold change ≥ 2.0, were subjected to MSig-DB analysis. The top up- and down-regulated gene signatures of Hallmark, Gene Ontology, Genetic and chemical perturbations, and Canonical pathways are presented. (**C**) Heatmap representation of the relative expression of known phenotypic markers for each of the indicated cellular processes.

EGCG reduces Notch and CBFA2T3 pathways in PMA-differentiated promyelocytic macrophage-like cells. We next conducted additional RNA-Sequencing analysis and profiled HL60 cells upon co-treatment with PMA and EGCG. We assessed the up- and down-regulated genes using Molecular Signatures DB (MSig-DB; Figure 4A,B) and found that EGCG-treated macrophage-like cells were inversely associated with stem-like features. Indeed, pluripotency (Plurinet) and embryonic stem cells gene signatures were decreased, while cell differentiation was significantly enriched (Figure 4A,B). Accordingly, reduced levels of key regulatory genes involved in the promotion of stemness and pluripotency (*LSM4*, *LSM5*, and *HSPE1*), and enhanced levels of differentiation genes (*BMP10* and *SLIT2*) were observed in the EGCG-treated macrophage-like cells (Figure 4C). This was accompanied by reduced Notch signaling, a critical regulating pathway of hematopoietic stem cells [35] (Figure 4A). This was corroborated by the repression of the transcript level of histones (*H4C8*, *H4C9*, *H2BC5*, and *H2BC21*) and the mammalian target of rapamycin (mTOR) activator/regulator complex LAMTOR4 (Figure 4C).

Surprisingly, we also observed a reduction in the CBFA2T3 pathway upon EGCG treatment. The CBFA2/RUNX1 partner transcriptional corepressor 3 (CBFA2T3, also known as MTG16 or ETO2) acts as a transcriptional corepressor to regulate hematopoietic stem/progenitor cell proliferation and lineage allocation [36] (Figure 4A). To our knowledge, the capacity of EGCG to downregulate *CBFA2T3* has never been described before. Negative enrichment for the CBFA2T3 pathway, associated with the transcriptional reduction in *CBFA2A3*, *MS4A3*, *PRG2*, and *PRTN3* transcripts, was observed in the EGCG-treated promyelocytic macrophage-like cells (Figure 4C).

EGCG treatment of macrophage-like cells was also found to increase apoptosis and response to stress gene signatures (Figure 4B). Positive enrichment in immune function, including induced natural killer T cells, as characterized by increased *CCL5*, *CSTB*, and *IFIT3* transcripts levels, was also observed in EGCG-treated macrophage-like cells (Figure 4C). These results indicate that EGCG induces a transcriptomic program characterized by reduced proliferative and stem-like features, including the downregulation of Notch and CBFA2T3 pathways.

**Figure 5:**
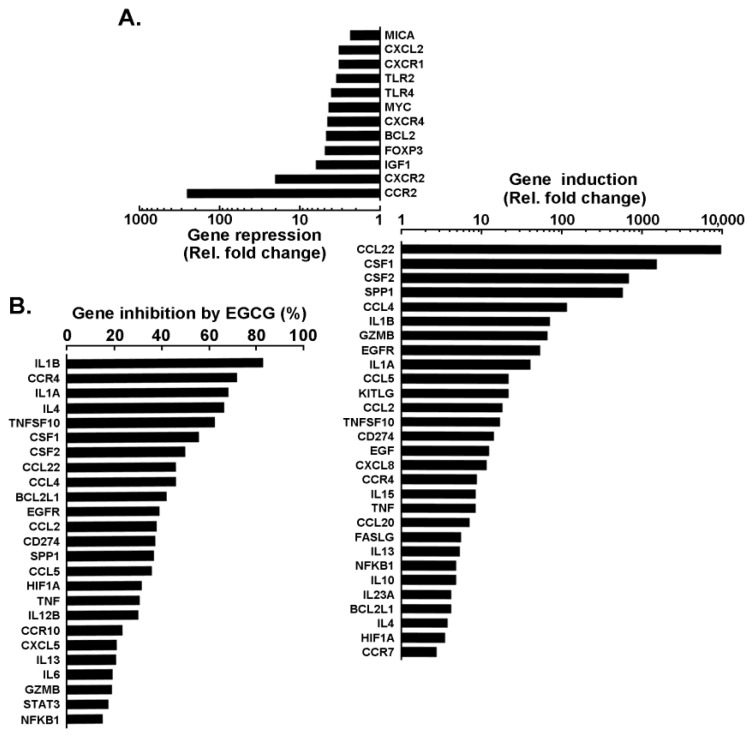
Impact of EGCG on PMA-mediated transcriptional regulation of immunity and inflammation biomarkers expression. Total RNA was isolated from human HL60 cells in suspension or adherent cells resulting from PMA treatment in the absence or presence of 30 μM EGCG. Reverse transcription was performed to generate cDNA as described in the Methods section and results from gene arrays are presented in (**A**) for the top 30 genes that were downregulated (**top**) or upregulated (**bottom**) and in (**B**) for the extent of EGCG inhibition.

Impact of EGCG on PMA-mediated transcriptional regulation of immunomodulatory and inflammation biomarkers expression. Finally, the transcriptional crosstalk between immunomodulatory and inflammatory genes reflecting the molecular signature of PMA-differentiated promyelocytic macrophage-like cells was explored. Total RNA was isolated from human HL60 cells in suspension or adherent cells resulting from PMA treatment in the absence or presence of 30 μM EGCG. Reverse transcription was performed to generate cDNA as described in the Methods section and results from gene arrays were presented for the top 30 genes that were downregulated (Figure 5A, top) or upregulated (Figure 5A, bottom). The extent of EGCG inhibitory impact was also plotted. Interestingly, *CCL22*, *CSF1*, *CSF2*, *CCL4*, *IL1B*, *CCL2*, *TNF*, among other genes, were found to be induced from 10–10,000 times and efficiently inhibited by EGCG (Figure 5B).

## 4. Discussion

The phenotypic polarization of tumor-associated macrophages (TAM) and the development of tumours are influenced by the interactions between invading macrophages, tumour cells, and the stromal microenvironment [4]. As a result of their enhanced antitumor inflammatory responses, M1 phenotype macrophages are resistant to tumours [36], whereas M2 macrophages, also known as TAM, are endowed with a variety of tumor-promoting abilities including, among others, immuno-suppression, angiogenesis, as well as stromal activation and remodeling [37]. Here, we show that diet-derived anti-inflammation and anticancer EGCG prevents the PMA-mediated signaling that triggers macrophage-like differentiation. Interestingly, such chemopreventive effects were recently found to block macrophage polarization from M1 to M2 in bone marrow-derived macrophages [38] and to modulate polarized macrophages by suppressing M1 phenotype and promoting M2 polarization in vitro and in vivo [39].

Here, we exploited the well-established PMA-mediated HL60 macrophage-like differentiation model to explore the phenotypic crosstalk that could link immune-suppressive and inflammatory molecular signatures [12]. Most importantly, we also addressed whether diet-derived polyphenol-mediated intervention may prevent the acquisition of such phenotype. Plants contain large amounts of polyphenols, which are known to have a variety of anticarcinogenic properties. These include the ability to prevent the growth of tumours, angiogenesis, metastasis, and inflammation as well as to cause apoptosis. Additionally, they can control immunological response and shield healthy cells from free radical deterioration [40]. The immune system has both beneficial and detrimental impacts on carcinogenesis, and the inflammatory milieu is a crucial part of tumours accordingly [41,42,43]. Given its demonstrated ability to regulate several molecules, including inflammatory cytokines, chemokines, growth factors, reactive oxygen and nitrogen species and to target the chronic inflammation process. Then, it is reasonable to hypothesize that EGCG may consequently also alter transcriptomic programs involved in the immune phenotype and which could then also contribute to triggering the tumor angiogenesis and inflammation. PMA-triggered cell differentiation induces cell-cycle arrest as well as elevated expression of macrophage markers CD11b, CD13, CD14, CD45, EGR1, CSF1R, and IL-8. PMA also increased nuclear translocation of autophagy transcription factors TFEB, FOXO1, and FOXO3, as well as the expression of several autophagy-related (ATG) genes in HL60 cells [44].

The main polyphenol found in green tea is EGCG. By reducing the expression of iNOS and COX-2 genes, it has been consequently demonstrated to also decrease the synthesis of pro-inflammatory mediators such as NO and PGE2 [10,45]. Besides EGCG, immunomodulatory therapeutic effects of other diet-derived molecules such as curcumin on M1/M2 macrophage polarization in inflammatory diseases have been reported. In addition, and similarly to EGCG, curcumin inactivation of FoxM1 was found to impact its downstream genes including, MMP-9 and vascular endothelial growth factor, leading to the reduction in survival and angiogenesis, and enhanced chemosensitivity in AML [46]. Pathologic inflammatory conditions are frequently correlated with dynamic alterations in macrophage activation with curcumin anti-tumor immunity properties to shift M polarization status and to act as a potential modulator of M1 and M2 macrophages [47,48]. While macrophages isolated from primary tumors display an anti-inflammatory M2 phenotype and/or M2 polarized by the tumor cells, diet-derived intervention may prevent their capacity to escape their destruction by the immune system possibly by inducing apoptosis or cell senescence [49].

Here, we also exploited EGCG’s kinase inhibitory activity through a phosphokinase array and found that MSK, RSK, CREB, and GSK phosphorylation status was all triggered upon PMA-mediated differentiation of HL60 cells into adherent macrophage-like cells. Strategies have been previously employed to modify the macrophage polarization status as the repolarization of M2 TAM would play an essential role in anti-cancer therapy [50,51]. Among the signaling intermediates identified above, the CREB pathway is known to link PGE2 signaling with macrophage polarization [52]. In fact, a CREB-C/EBPβ cascade has been shown to induce M2 macrophage-specific gene expression [53], and was higher in M2 polarized cells [54]. M2 polarization also correlates with a predominant arginase pathway and an increased production of polyamines, which can be responsible for tumor proliferation together with an anti-inflammatory response [55]. Interestingly, EGCG was found to inhibit arginase [56], but whether this contributes to reducing TAM’s association with angiogenesis and metastasis in tumors remains unknown [57]. Intriguingly, EGCG was unable to inhibit PMA-induced CREB phosphorylation, which does not preclude a potential indirect action on downstream arginase activity. However, the pleiotropic capacity of EGCG to inhibit ERK/MSK/RSK/GSK signaling intermediates confirm its multi-kinase targeting and chemopreventive activities.

Here, we further report, for the first time to our knowledge, the ability of EGCG to downregulate the CBFA2T3 (MTG16) pathway. CBFA2T3 is a transcriptional corepressor upregulated in hematopoietic stem and progenitor cells [58]. The rapid expansion of short-term stem cells, multipotent progenitor cells, and megakaryocyte-erythroid progenitor cells, which are necessary during hematopoietic stress/emergency, is hampered by *Mtg16* gene inactivation [35]. According to a number of studies, CBFA2T3 inhibits differentiation and enhances proliferation in a range of hematopoietic cell types [59,60,61,62]. Targeting CBFA2T3 in leukemia, especially acute lymphoblastic leukemia that expresses the E2A-Pbx1 fusion protein, is also supported by an increasing body of evidence [58,62,63].

In terms of potential translational implications for AML in support to EGCG’s capacity to inhibit differentiation processes, the closest molecular rationale one can provide at this point is that EGCG killing capacity appears to be closely associated with higher expression of the 67 kDa laminin receptor (67LR) in AML patient samples and differentiating AML cells than in controls [64]. Moreover, 67LR is overexpressed in 40% of de novo AML but is undetectable in normal bone marrow hematopoietic cells [65]. Interestingly, 67LR is a critical cell-surface mediator for EGCG pharmacology [66]. Thus, low 67LR expression in normal cells and its overexpression in transformed cells could not only be of use as a predictive marker for the efficacy of EGCG treatment, but moreover provide a molecular explanation for the specific activity of EGCG on cancer cells. Interestingly, 67LR was found reduced in terminally differentiated HL60 macrophages [16], re-enforcing the concept explored herein regarding the chemopreventive targeting efficacy of EGCG against HL60 differentiation processes. Interestingly, recent studies found high 67LR expression in CD34-positive AML cells compared to normal CD34-positive cells and could show that high 67LR expression possibly contributes to a poor prognosis of these AML patients [67]. One might speculate that these patients with high 67LR expression, suffering from a highly proliferative and apoptosis-resistant blast cell population, would most efficiently benefit from EGCG treatment.

## 5. Conclusions

We do acknowledge that the sole use of the well-documented HL60 cell line model may preclude robust conclusions as every cell line has different characteristics. Nevertheless, the present study revealed EGCG’s ability to prevent the acquisition of an immunosuppressive and inflammatory phenotype through the inhibition of multiple signal transducing events. Whereas this may cause the decrease in certain polarization markers only, or not a full repolarization from M1 to M2 phenotype or vice versa, this still supports the pleiotropic chemopreventive properties of diet-derived polyphenols.

## Data Availability

All data generated or analyzed during this study are included in this published article.

## Supplementary Materials

The following supporting information can be downloaded at: https://www.mdpi.com/article/10.3390/cancers14205065/s1, File S1. Full pictures of the Western blots for Figure 3.

## Abbreviations

AML, Acute myeloid leukemia; CDK, Cyclin-dependent kinase; CREB, cAMP response element-binding protein; EGCG, Epigallocatechin-3-gallate; FC, Fold change; FDR, False discovery rate; GO, Gene ontology; GSEA, Gene set enrichment analysis; GSK, Glycogen synthase kinase; HuR, Human antigen R; 67LR, 67 kDa laminin receptor; MMP-9, Matrix metalloproteinase-9; MSK, Mitogen and stress activated protein kinase; PMA, Phorbol 12-myristate 13-acetate; PKC, Protein kinase C; RSK, Ribosomal s6 kinase; TAM, Tumor-associated macrophage.
