# Supplementary material for: EGCG Prevents the Transcriptional Reprogramming of an Inflammatory and Immune-Suppressive Molecular Signature in Macrophage-like Differentiated Human HL60 Promyelocytic Leukemia Cells
Source: Cancers (Basel). 2022 Oct 16;14(20):5065. doi: 10.3390/cancers14205065 (PMC9599716; doi:10.3390/cancers14205065)
Supplement: Supplementary file 1 [file cancers-14-05065-s001.zip › File S1. WB figure 3 article CK 1.pptx]

## Slide 1
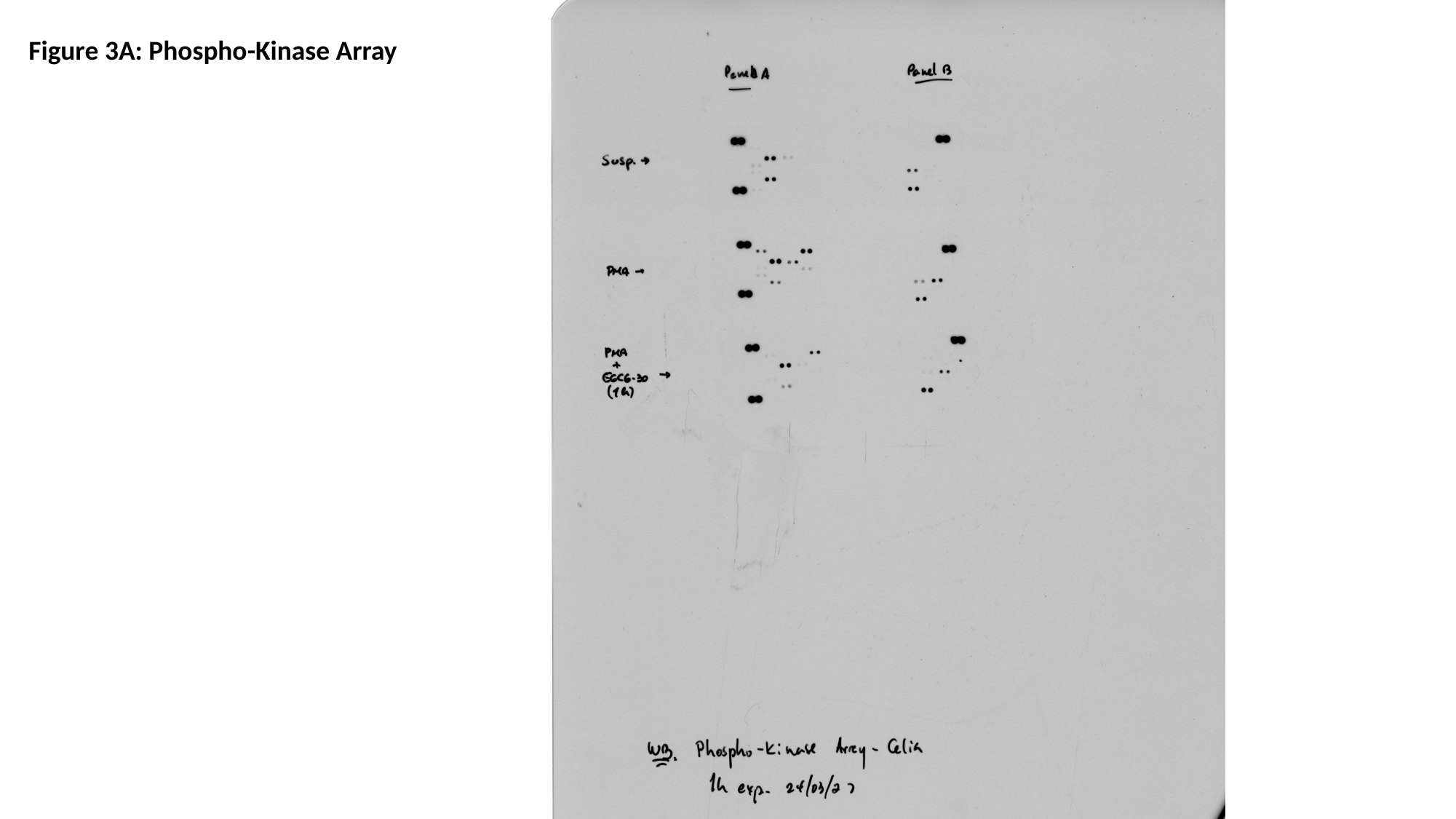

Figure 3A: Phospho-Kinase Array

## Slide 2
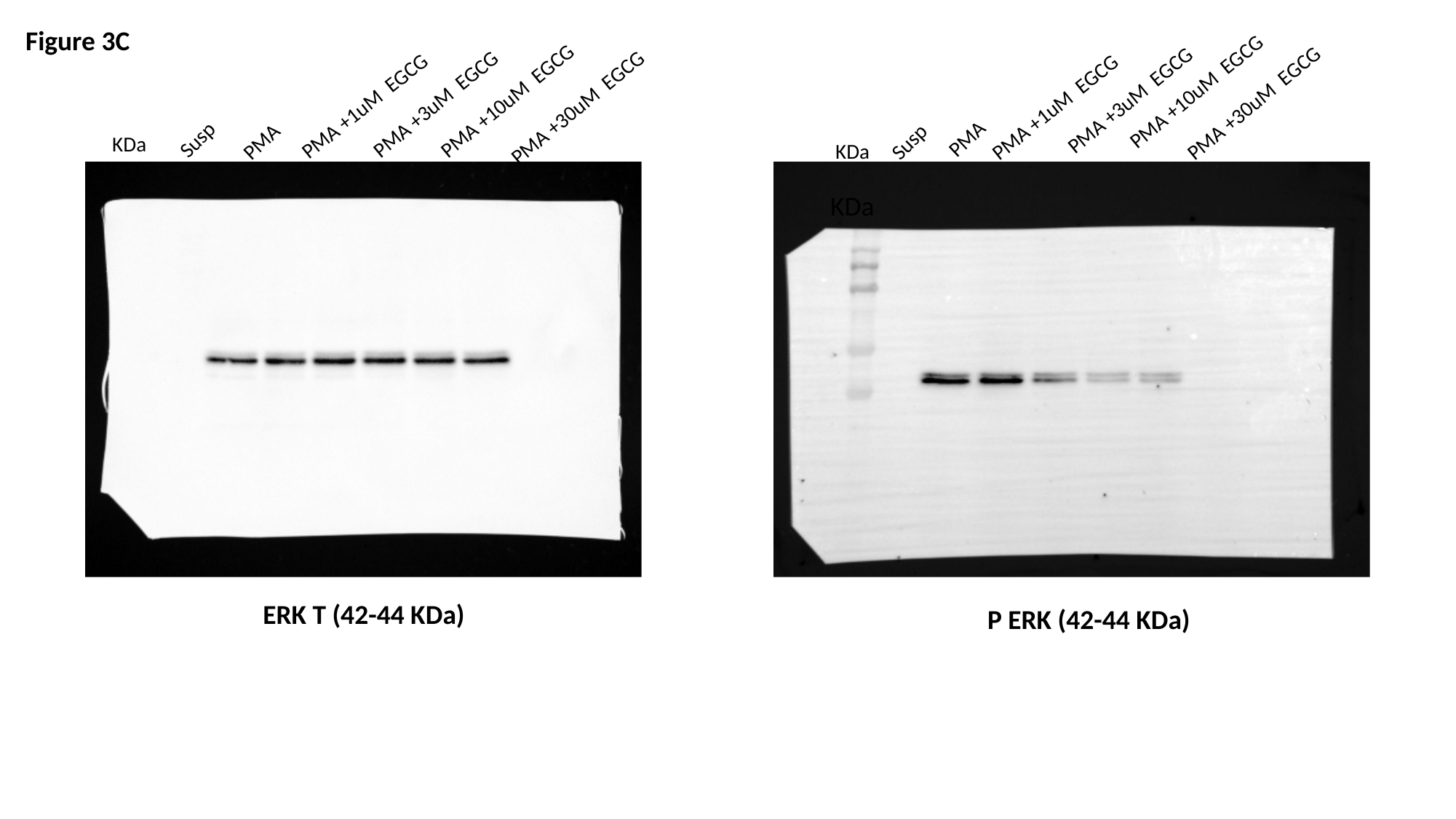

Figure 3C
PMA +10uM EGCG
PMA +3uM EGCG
PMA +10uM EGCG
PMA +3uM EGCG
PMA +30uM EGCG
PMA +1uM EGCG
PMA +1uM EGCG
PMA +30uM EGCG
PMA
PMA
Susp
Susp
KDa
KDa
KDa
ERK T (42-44 KDa)
P ERK (42-44 KDa)

## Slide 3
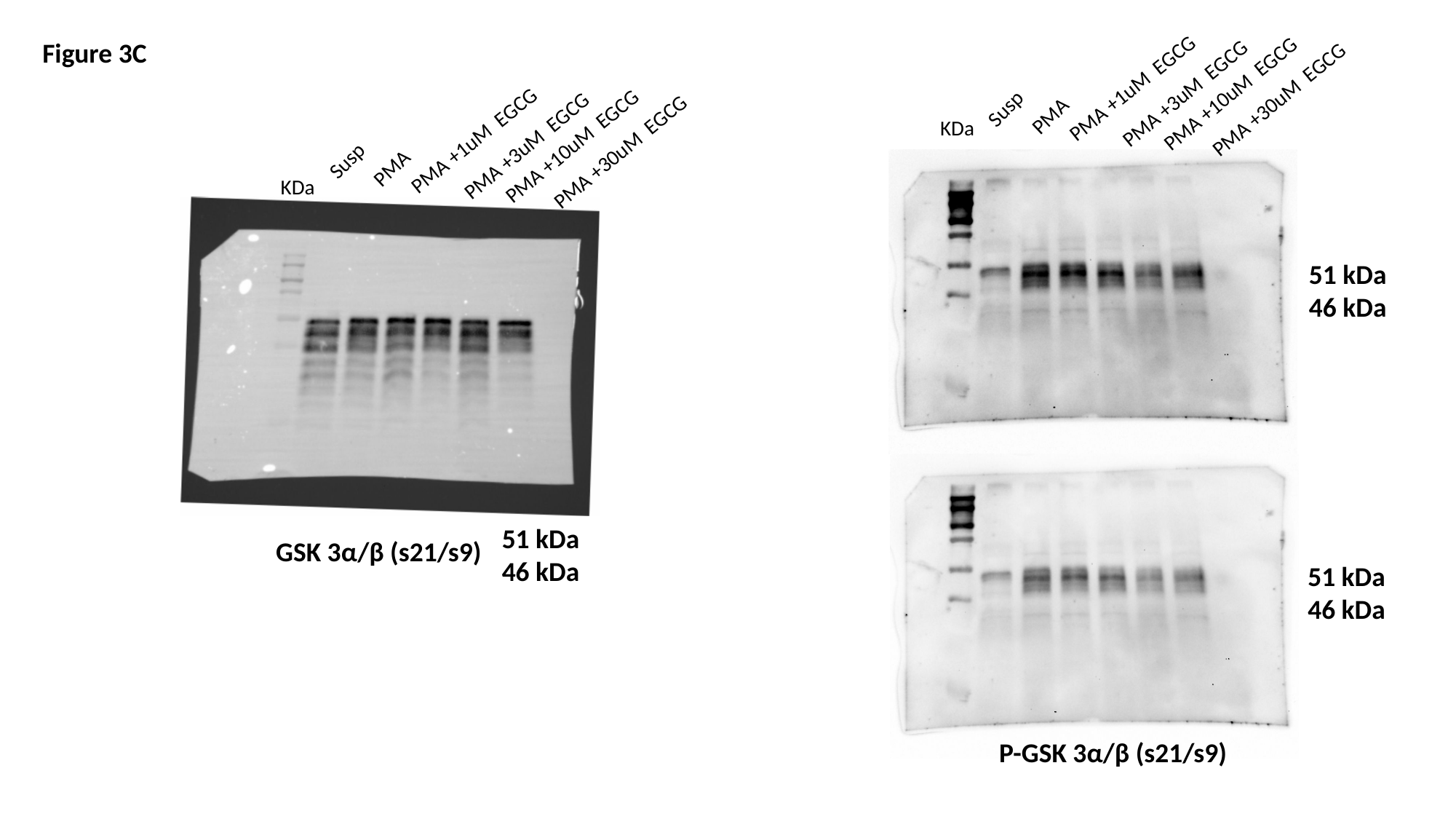

Figure 3C
PMA +1uM EGCG
PMA +3uM EGCG
PMA +10uM EGCG
PMA +30uM EGCG
Susp
PMA
PMA +1uM EGCG
PMA +3uM EGCG
KDa
PMA +10uM EGCG
PMA +30uM EGCG
Susp
PMA
KDa
51 kDa
46 kDa
51 kDa
46 kDa
 GSK 3α/β (s21/s9)
51 kDa
46 kDa
P-GSK 3α/β (s21/s9)

## Slide 4
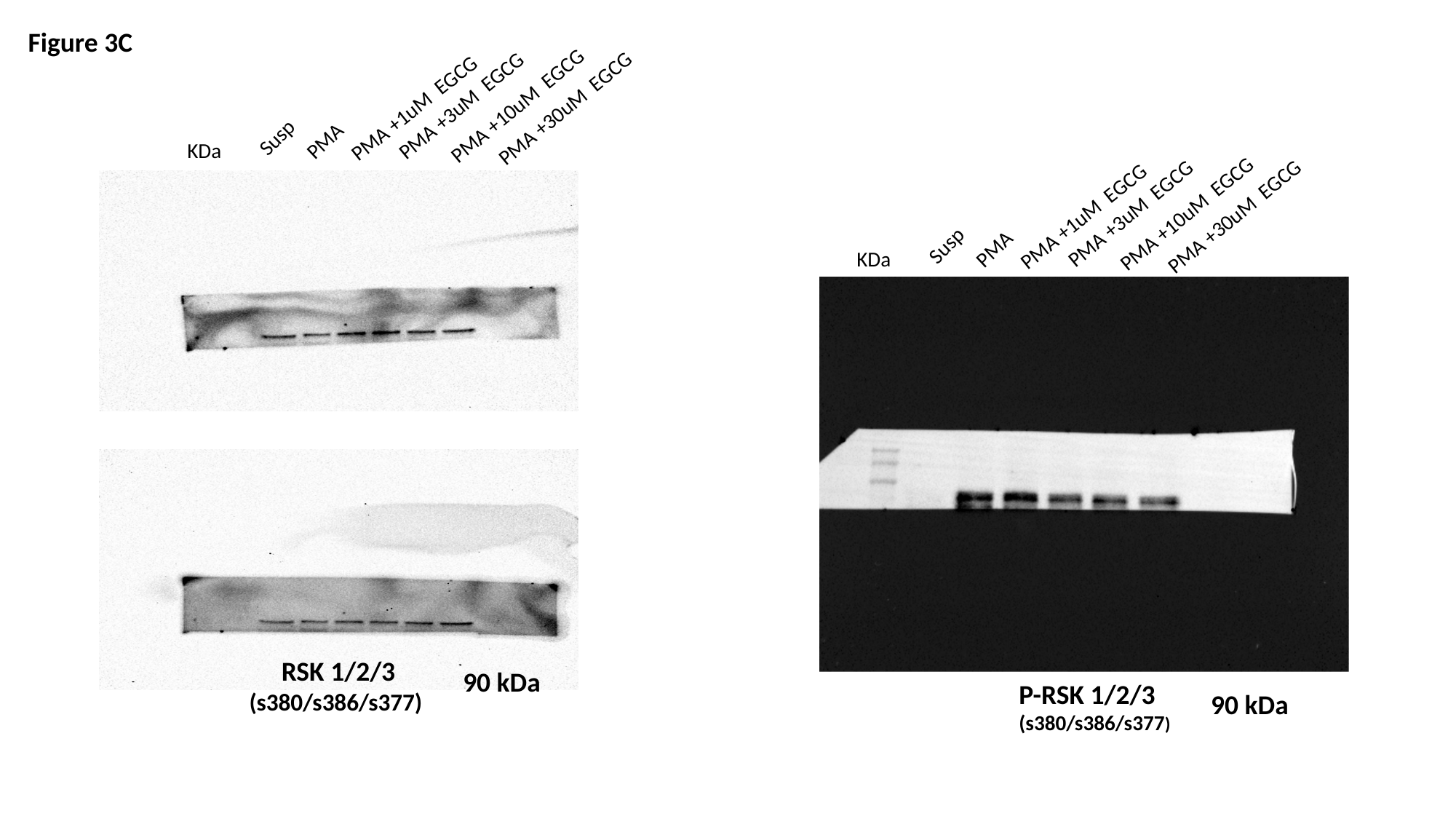

Figure 3C
PMA +3uM EGCG
PMA +10uM EGCG
PMA +30uM EGCG
PMA +1uM EGCG
PMA
Susp
KDa
PMA +3uM EGCG
PMA +10uM EGCG
PMA +30uM EGCG
PMA +1uM EGCG
PMA
Susp
KDa
 RSK 1/2/3
 (s380/s386/s377)
90 kDa
P-RSK 1/2/3 (s380/s386/s377)
90 kDa

## Slide 5
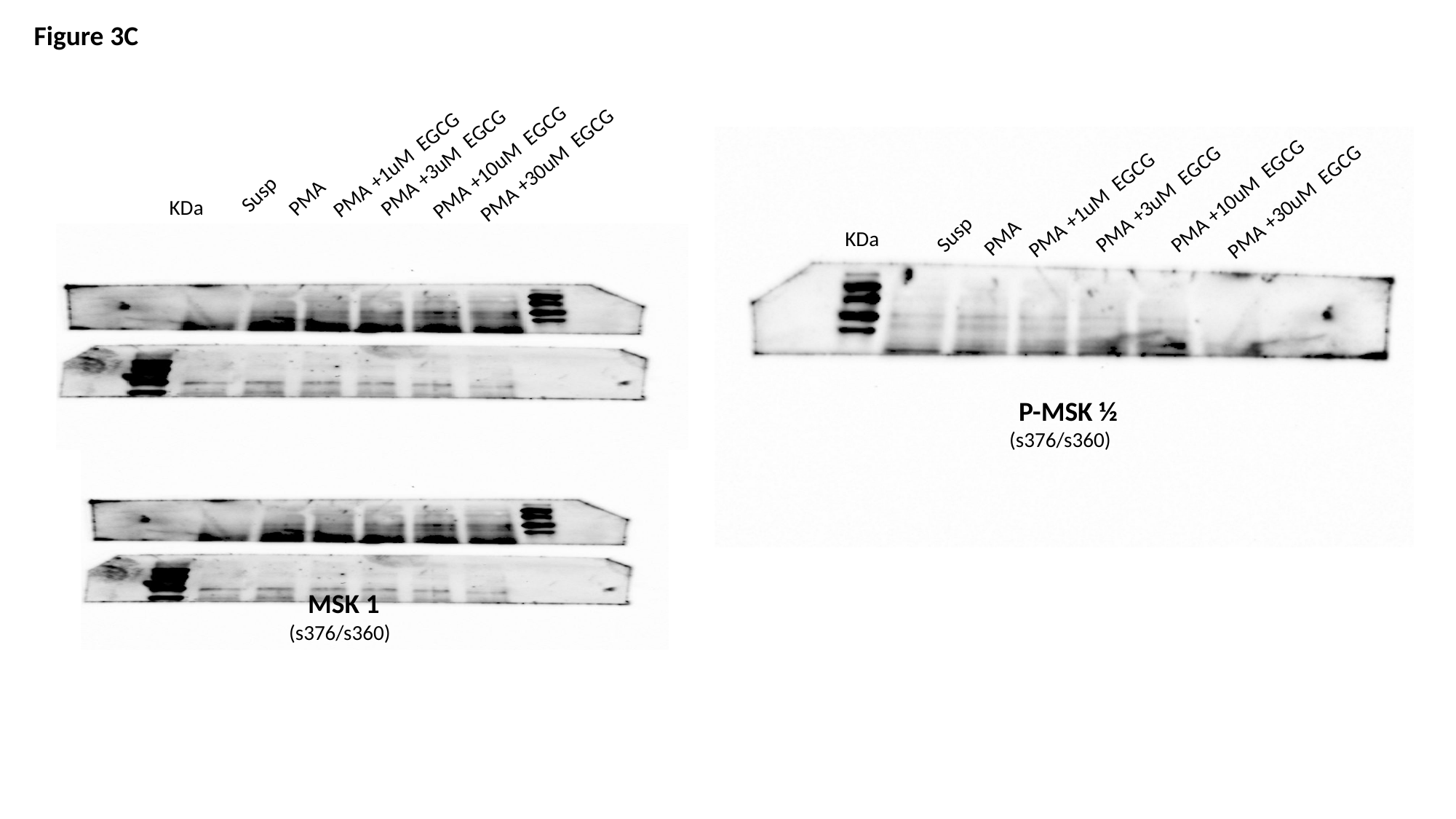

Figure 3C
PMA +3uM EGCG
PMA +10uM EGCG
PMA +30uM EGCG
PMA +1uM EGCG
PMA +10uM EGCG
PMA +3uM EGCG
PMA
Susp
PMA +30uM EGCG
PMA +1uM EGCG
KDa
PMA
Susp
KDa
 P-MSK ½
(s376/s360)
 MSK 1
(s376/s360)

## Slide 6
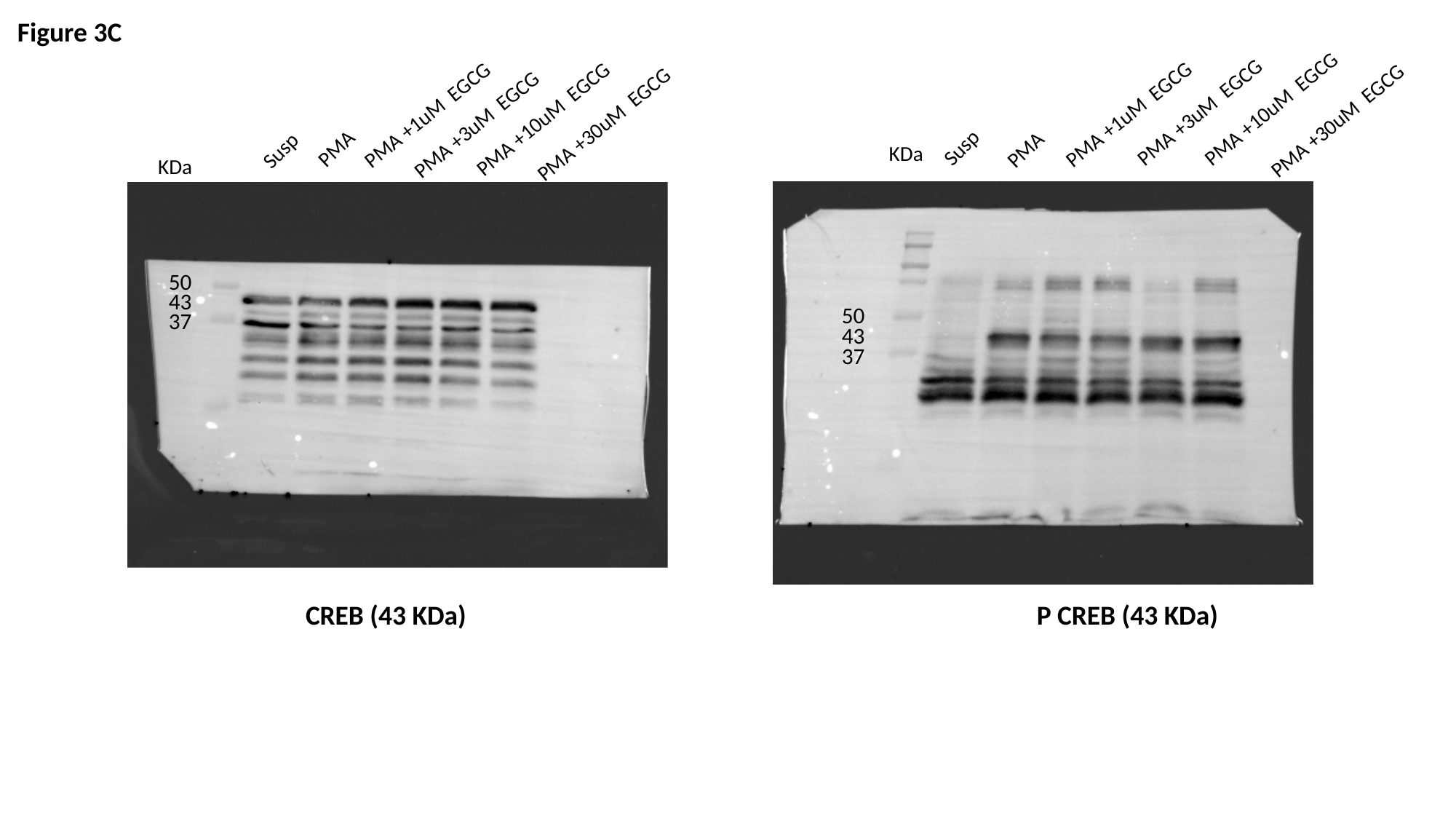

Figure 3C
PMA +10uM EGCG
PMA +3uM EGCG
PMA +1uM EGCG
PMA +1uM EGCG
PMA +10uM EGCG
PMA +30uM EGCG
PMA +3uM EGCG
PMA +30uM EGCG
PMA
PMA
Susp
Susp
KDa
KDa
50
43
50
37
43
37
CREB (43 KDa)
P CREB (43 KDa)
